# Healthcare seeking practices and barriers to accessing under-five child health services in urban slums in Malawi: a qualitative study

**DOI:** 10.1186/s12913-016-1678-x

**Published:** 2016-08-19

**Authors:** Edgar Arnold Lungu, Regien Biesma, Maureen Chirwa, Catherine Darker

**Affiliations:** 1 Department of Planning and Policy Development, Ministry of Health, P.O Box 30377, Lilongwe, Malawi; 2 Department of Epidemiology and Public Health Medicine, Royal College of Surgeons in Ireland, Lower Mercer Street, Dublin 2, Ireland; 3 Prime Health Services and Consultancy, Area 47 Sector 4, Lilongwe, Malawi; 4 Department of Public Health & Primary Care, Trinity College Dublin, Tallaght Hospital, Dublin 24, Ireland

**Keywords:** Under-five children, Health system, Urban slums, Healthcare-seeking

## Abstract

**Background:**

Access to child health services is an important determinant of child health. Whereas, child health indicators are generally better in urban than rural areas, some population groups in urban areas, such as children residing in urban slums do not enjoy this urban health advantage. In the context of increasing urbanisation and urban poverty manifesting with proliferation of urban slums, the health of under-five children in slum areas remains a public health imperative in Malawi. This paper explores healthcare-seeking practices for common childhood illnesses focusing on use of biomedical health services and perceived barriers to accessing under-five child health services in urban slums of Lilongwe, Malawi’s capital city.

**Methods:**

Qualitative data from 8 focus group discussions with caregivers and 11 in-depth interviews with key informants conducted from September 2012 to April 2013 were analysed using conventional content analysis.

**Results:**

Whereas, caregivers sought care from biomedical health providers, late care-seeking also emerged as a major theme and phenomenon. Home management was actively undertaken for childhood illnesses. Various health system barriers: lack of medicines and supplies; long waiting times; late facility opening times; negative attitude of health workers; suboptimal examination of the sick child; long distance to health facility; and cost of healthcare were cited in this qualitative inquiry as critical health system factors affecting healthcare-seeking for child health services.

**Conclusions:**

Interventions to strengthen the health system’s responsiveness to expectations are essential to promote utilisation of child health services among urban slum populations, and ultimately improve child health and survival.

**Electronic supplementary material:**

The online version of this article (doi:10.1186/s12913-016-1678-x) contains supplementary material, which is available to authorized users.

## Background

The Millennium Development Goals (MDGs) significantly shaped global and national health policy content and child health discourse for the past decade. The Malawi MDG end line survey reports that the country has achieved its MDG four [1], which aspired to reduce under-five mortality rate by two-thirds by 2015 from the 1990 levels [2]. The MDG 4 target for Malawi entailed an under-five mortality rate of 78 deaths per 1000 live births, which should still be a cause for concern.

It was widely agreed that attainment of MDGs needed to have equity considerations for improvements to be sustainable and in respect of the right to health for all [3]. Arguably, child health indicators will show dismal improvements if the health of children in disadvantaged settings stagnates. To meaningfully improve child health indicators also entails tremendous efforts to increase access to health services among the worse-off groups. Moreover, the newly adopted Sustainable Development Goals succeeding the MDGs aspire for equitable and sustainable development. For example, SDG 3 makes direct reference to health and states the need to ‘Ensure healthy lives and promote well-being for all at all ages’. Specifically, target 3.1 asserts: “End preventable deaths of new-borns and children under 5 years of age, with all countries aiming to reduce neonatal mortality to at least as low as 12 per 1,000 live births and under-5 mortality to at least as low as 25 per 1,000 live births” [4].

In Malawi, as much as 50 % of under-5 child deaths occur in the community, some without contact with a health facility [5], reflecting poor healthcare-seeking behaviours. However, this represents aggregate levels which mask differentials among populations. In recent years, the health disadvantage of populations residing in urban slums with regard to child health outcomes and health service uptake, has been subject of policy discussion, but for which there is limited evidence in some settings [6]. Some authors observe that large demographic and health surveys hardly focus on the urban poor and slum areas hence not providing information on a significant aspect of urban health [7, 8]. In the context of Malawi being one of the fastest urbanising countries, with an urbanisation rate of 6.2 % which is twice the African rate of urbanisation of 3.2 % and an increasing population residing in slum conditions [9], this presents a crucial public health issue warranting attention, including improving access to child health services.

The evidence that access to healthcare is a crucial determinant of health, including child health, has existed for some time and continues to emerge. The Knowledge Networks on Urban Settings (KNUS) and Health Systems (KNHS) of the World Health Organisation’s Commission on Social Determinants of Health posit that health systems promote population health independent of other influences [10, 11]. Access to healthcare has been shown to reduce child mortality and morbidity [12–14].

Like many developing countries, children in the urban areas in Malawi have better geographical access to healthcare services relative to their rural counterparts [7, 15]. However, little is known about experiences of caregivers from urban slums when seeking under-five child healthcare services despite the increasing importance of this group in the context of rapid urbanization rate. While Malawi’s slums are not comparable in both size and deprivation levels to those of big cities in other developing countries, there are settings in urban areas in Malawi that befit the slum classification and face worse health outcomes than other urban residents.

A slum is classified as an urban geographical entity lacking in *one or more* of the following five conditions: access to improved water, access to sanitation, durable housing, sufficient living area, and secure tenure [16]. Each of these characteristics is a crucial determinant of health and specific risk factors for childhood malaria, diarrhoea, Acute Respiratory Infections (including pneumonia) which represent commonest childhood conditions in Malawi [6].

The UN-HABITAT estimates that 61 % of the population in Malawi’s capital city Lilongwe resides in slum conditions [9]. Whilst there is dearth of epidemiological evidence on the burden of childhood conditions in Malawi’s urban slums, evidence from elsewhere suggests that child health indicators are worse off in urban slums than in non-slum areas and even in rural areas [8, 17, 18]. For example, the African Population and Health Research Centre has demonstrated that child mortality in Nairobi slums is much higher than the rural areas of Kenya and the national average [8]. In such contexts, under-five children constitute a vulnerable population group deserving public health attention including enhancing access to healthcare. On this basis, this study’s focus on access to health for children in urban slums was considered worthwhile for child health programming in Malawi. The study will unearth important dynamics of decision making pertaining to use of biomedical child health services and inform how the health system can be better organised to be responsive to clients’ expectations, subsequently promoting utilisation of essential child health services and ultimately improve the health and survival of children in urban slums.

Some previous studies on healthcare utilisation in Malawi and elsewhere, [12, 19, 20] have mostly used quantitative designs, limiting the depth of expressions, rich thematic texture and exploratory information inherent in qualitative studies. [21]. This qualitative study was conducted to explore healthcare-seeking practices for common childhood illnesses focusing on use of biomedical health services and perceived barriers to accessing under-five child health services in urban slums of Lilongwe, Malawi’s capital city.

## Methods

### Design

This was a qualitative study using Focus Group Discussions (FGDs) with care givers of under-five children and In-depth interviews with key informants in child health service delivery. It was part of a larger longitudinal study on child health and survival in urban slums. Findings of this study were important in defining health system attributes to be quantitatively explored in the larger study, with regard to their relative importance in influencing utilisation of child health services among urban slum care givers.

### Setting

In the Malawi health system, health services are predominantly delivered by the public sector (free at the point of use), Christian Health Association of Malawi (CHAM – which is an umbrella body of Christian faith based health facilities operating on a not-for-profit basis); the private health sector (which charges user-fees); and the NGO sector [5]. Public and CHAM health facilities constitute two largest providers, collectively providing about 90 % of health services [22] in urban and rural areas.

The study was conducted in three randomly selected urban slums in Lilongwe, the capital city and fastest urbanising of Malawian cities [5]. The random selection process entailed two steps. Firstly, based on UN-HABITAT’s urban slum definition [16] and consultations with experts from the local UN-HABITAT and City Assembly, all urban slum areas in Lilongwe were identified. Secondly, a simple random selection was used that involved placing pieces of paper with names of identified slum areas written on them in a bowl, shaking it and randomly picking three pieces of paper. This allowed for each slum area to have equal opportunity to be selected to become a study site. To protect the confidentiality of participants study sites are referred to as Site 1, Site 2 and Site 3.

### Participant selection

Participants were selected using non-probability sampling method of convenient sampling technique. The procedure for selecting FGD participants entailed going door-to-door and selecting eligible study participants who were available at the time of recruitment. However, an attempt was made to select participants from different localities within a particular urban slum by recruiting over a geographically wide area. The inclusion criteria for participation in FGDs entailed the following: residence in study setting, present or past experience of being a primary care giver of an under-5 year old child, not participated in the pilot interview and provision of consent to participate

Snowball sampling, which involves recruiting key informants identified through other initial study participants [23] who were purposively selected, was used to select participants for in-depth interviews. Purposive selection of the initial study participants followed an eligibility criterion for key informants that entailed current involvement in issues of child health at both policy and service delivery levels. In this regard central level child health programme managers, district health managers and health workers involved in child health service delivery were selected at the initial stage, informed by the corresponding authors (EL) understanding of the health system context and consultations with officers from Ministry of Health and District Health Officer on institutional entry for this study.

The selection of both service users (care givers in FGDs) and health providers (policy makers and health workers in KII) was deemed useful for purposes of obtaining all relevant perspectives in context of our study thereby providing balanced information and a more comprehensive understanding of the phenomena under study.

A saturation point, defined as the phenomenon in which additional sampling units are not yielding new information, [23], was used to determine the ultimate sample size. Consequently, 8 FGDs (two in Site 1 and three each in Sites 2 and 3) were conducted. Each FGD involved 10 to 12 participants, and collectively, 89 caregivers participated in FGDs. A total of 11 participants were involved in In-depth Key Informant Interviews (KII) hence the full sample constituted 100 participants (see Table 1).

**Table 1:** Sample of FGD and KII

Sampling unit	Number sampled
Key Informant Interview (KII) Participants	
Health policy	1 (National Coordinator of one of the child health programmes)
City Council	1 (One of the coordinators of health at Lilongwe City Council)
District Health Office (District Management)	1 (One senior District Health Management Team member)
Health workers at facility level	4 (2 medical assistants and 2 nurses)
Health workers at community level	3 Health Surveillance Assistants (HSAs), one in each study area
UN-HABITAT	1
Focus Group Discussions	
Site 1	2 (FGD 1: *N* = 10; FGD 2 *N* = 12)
Site 2	3 (FGD 1: *N* = 12; FGD 2 *N* = 11; FGD 3 *N* = 12)
Site 3	3 (FGD 1 *N* = 12; FGD 2 *N* = 10; FGD 2 *N* = 10)

### Data collection

Prior to undertaking discussions and interviews, informed verbal consent (including for audio recording) was obtained for voluntary participation in the study. Interview guides for both FGD and KIIs were piloted among eligible participants for FGDs and KIIs and the data gathered was not used in analysis but was useful to inform relevant modifications in content of the tools and interviewing techniques. Piloting for FGDs was conducted in one study setting (site 3) and participants of this pilot did not participate in the main study. Pretesting of the KII tool was conducted among health workers in a health facility outside the study setting but serving a similar population group in another slum area. Following pretesting, both FGD and KII tools were modified accordingly and used for data collection. Focus group discussion guide and KII interview guide have been provided as Additional files 1 and 2.

Owing to flexibility of qualitative research, the interview guides were also modified based on information emerging in the course of data collection. However, the overarching issues in the interview guides focussed on the following areas: responses to childhood illnesses, timely (or conversely late) care-seeking from a biomedical health provider, barriers to under-five child healthcare use.

Data collection was conducted from September 2012 to April 2013. All the 8 FGDs were conducted by the corresponding author with an assistant in local Chichewa language. The corresponding author also conducted all KII. Four (4) KII were conducted with community health workers in Chichewa whereas policy and district management level KII were conducted in English. Participants in FGDs and KIIs were encouraged to engage in free flow discussions. All FGD and KII interview sessions were recorded using a digital voice recorder. On average, FGDs took approximately 60 min and KII took 35 min.

We defined late care-seeking as seeking child healthcare services after 24 h of onset of childhood symptoms. This was based on the African Union health resolution that every child with fever should seek care from a health provider within 24 h [24]. Considering the measurement difficulties of’24 h’ in a setting of low literacy levels, we descriptively referred to a period from onset of symptoms to next day. Thus, after two days best described beyond 24 h from onset of symptoms.

All FGD and KII audio recordings were transcribed verbatim by trained research assistants and those conducted in local Chichewa language were then translated to English by the corresponding author and a secondary school teacher who taught English language and communication.

### Data analysis

Conventional content analysis involving the identification, coding and categorisation of primary patterns in data and ultimately drawing meaningful relationships [25] was used for this study. Conventional content analysis is generally used to describe a phenomenon, in this case, care seeking practices and barriers to accessing under-five child health services. It allows for researchers immersion in the data for new insights to emerge [25]. After repeated reading of two FGD and three KII transcripts, two coding frames, one for KII and another for FGD transcripts were generated. The coding frames constituting super-ordinate themes and their supporting themes were developed by two authors with varying levels of immersion in the data. The corresponding author (EL) conducted all FGDs and KII, was involved in transcription, translation, and undertook detailed coding. Another author (CD) read and also coded the transcripts using the coding frames.

In the iterative process of reading and analysing the transcripts, additional themes emerged and informed modifications to the initial coding frames. The third version of the coding frames was subsequently used for content analysis of all transcripts.

As previously cited, separate coding frames were used for transcripts from KII and FGD, each having undergone the aforementioned process, and informing separate analysis for health workers and policy makers on the one hand and care givers on the other hand. This was necessary as the healthcare providers and policy makers provided experiential information that was different to that of care givers.

A rigorous line-by-line transcript coding for final analysis was undertaken and disagreements were discussed and resolved where possible. A high inter-coder reliability was achieved with 90 % agreement. To calculate inter-coder reliability, we enumerated all codes from each pair of transcripts that the two coders analysed using the coding frames. From the total codes allocated in transcripts we ascertained the number of similar codes attached to transcript content (agreed coding). We then calculated a proportion of agreement with all agreed upon coding as numerator and total number of codes allocated as a denominator.

Inter-coder reliability scores of 80 % have been proposed to be acceptable in qualitative analysis, albeit dependent on the goal of the study [26]. On this basis, the inter-coder reliability for this study falls within acceptable optimal scores and is ultimately indicative of the validity of the coding frame; shared interpretive understanding and a reliable analysis. Some authors have contested the use of inter-coder agreement scores in qualitative studies [27]. However, this process still has merits of reducing researcher bias and ascertaining the validity of a coding frame as an analytical tool. Moreover inter-coder reliability scores have been used in other qualitative studies [27, 28].

### Ethical considerations

Ethical approval was granted by the National Health Sciences Research Ethics Committee in Malawi and the Research and Ethics Committee of Trinity College Dublin, Ireland under approval numbers NHSRC 895 and 27201107 respectively. Verbal (for FGDs) and written (KIIs) consent was sought for participation in this study. The informed consent process entailed explaining the study information to participants and requesting for their autonomous decision on willingness to participate. Pseudonyms are used to anonymise respondents.

## Results

### Characteristics of study participants

Participants in the FGDs were all females which is consistent with socio-cultural norms in the study context where primary care givers of children are female. The mean age of FGD participants was 28 years and the majority were of primary level of education. On average, care givers participating in FGDs had 3 children and 65 % of care givers reported to have two children under 5 years of age. The majority (93 %) had at least one child under the age of 5 years under their care at the time of the FGD. The remainder (7 %) had their youngest child under 7 years of age hence had recent experience of caring for an under-5 year old child.

Of the 11 key informants for in-depth interviews, 7 (64 %) were females. The work experience of key informants ranged from 2–17 years.

### Responses to childhood morbidity and patterns of care-seeking

Major themes emerging from this study predominantly relate to care-seeking patterns and health system factors that influence care-seeking for under-five child health services in the context of an urban slum in Malawi.

Three aspects of response to childhood morbidity were discussed: home management of childhood illnesses; healthcare-seeking; and whether care was being sought timely.

### Home management of childhood illnesses

In all FGDs, caregivers mentioned various ways of home management of fever and diarrhoea but indicated that for pneumonia (locally identified as *chibayo* and recognised through two principal symptoms: difficulties with breathing and coughing), they do not undertake home management, rather just take the child to the hospital.

Home management of fever that emerged from all FGDs entailed: sponging the child with cold water; bathing the child with lukewarm water; removing clothes; and giving the child antipyretics such as Panadol or Cafemol, and in some cases a combination of both. Wide variations were noted with regard to dosage and frequency of taking the antipyretics, some indicating dosages appropriate for child age while others were higher dosages.

In all FGDs, the use of Oral Rehydration Therapy (ORT) was cited for home management of diarrhoea. Thanzi ORS, which is a locally branded Oral Rehydration Salt, that is subsidised and being actively promoted through social marketing was strongly cited in this regard.

“We give Thanzi. Sometimes it happens that you already have the ORS, it may have remained from the one you received from the hospital during the child’s previous diarrhoea episode, so we just take the same and use” (Mirriam; Site1 FGD 1)

Continued breast-feeding and giving water were cited as home management of diarrhoea. In some FGDs, caregivers mentioned the use of antibiotics such as Flagyl and Bactrim at home for management of diarrhoea. In all FGDs, local shops were cited as the commonest source of drugs used for home management, with friends and relatives, and small private clinics being other sources.

### Healthcare-seeking from a biomedical health provider and timeliness in care-seeking

A biomedical health provider was defined as any health facility offering “western” medicine. This definition was provided prior to discussions in FGDs and key informant interviews to achieve a mutual conceptual understanding. In all FGDs, caregivers revealed that despite numerous challenges they face at the hospitals, the majority still use them. This notwithstanding, the use of Over-the-Counter drugs to treat childhood illness was reported as a common occurrence whether the child is eventually taken to hospital or not. Typical responses to discussions pertaining to seeking child healthcare from a biomedical health provider were as exemplified below:

“Most of caregivers go to the hospital. Others just buy medicine and administer them at home, they are lazy to go to the hospital” (Martha; Site2 FGD 2)

Discussions around whether healthcare is sought timely for the sick child attracted differing assertions. Timely care-seeking was defined prior to discussions and interviews, as seeking healthcare within 24 h of symptom onset (see justification under procedure). While some FGDs predominantly indicated timely care-seeking, in most cases immediately the child exhibits symptoms or the next day, others suggested that caregivers play a watchful waiting approach, consequently delaying care-seeking:

“Many people wait, they think the child’s condition will get better and they buy Panadol and when it is getting worse they buy Bactrim before they go to the hospital when the condition worsens. Some wait even up to 3 days. There are few people in this area who go to the hospital immediately the child gets sick” (Chimwemwe; Site3 FGD 3)

The majority of health workers in KII indicated that in most cases caregivers seek child health services late, waiting up to 4 days at home. Some health workers further suggested that caregivers in such localities use traditional medicine as a first point of call.

“……yet on the other hand, they will have visited the traditional doctor who gives all sorts of reasons, they could have visited him three or four times before they decide to come to the hospital....or say until that child is almost unconscious and then they… say the hospital did not help us. But that’s because we are the last stop” (Uchizi; Medical doctor)

The use of alternative medicine is prevalent albeit the perception of providers differs. Evidently questions pertaining to whether traditional medicine is used for sick children attracted mixed assertions with the majority of caregivers in FGDs acknowledging availability of traditional healers in their area but that very few use them for child health. However, most caregivers in almost all FGDs indicated that there are elders in their communities who know traditional medicine and are sometimes the first point of contact.

“There are no traditional healers here but there are elderly people who know traditional medicine…. So sometimes when the child is sick, they tell you to use some herbs and it helps as the child gets better” (Wongani; Site1 FGD 2)

### Barriers and facilitative factors for access to under-five child health services

Various factors related to demand (care giver, household) and supply (health system) were cited as barriers (or conversely as facilitative) of seeking healthcare for under-five children. We discuss some of the salient factors that emerged.

### Lack of medicines and medical supplies

Inadequate supply of medicines and other medical supplies emerged as a recurring theme in FGDs and KII at both policy and service delivery levels with dominant reference to public health facilities. Caregivers claimed that as a consequence of inadequate supply or unavailability of drugs at the health facility, they are either given an incomplete dosage of prescribed medicines (for example one type of medicine instead of two that are prescribed and not necessarily fewer tablets of the same drug) or are not given at all.

“Sometimes the health facility does not have all medicines available and they end up giving inadequate drugs.…, we travel long distances to get there so if you consider going to a hospital under those circumstances you just get discouraged and stay at home. By the time you really have no choice but to go, the child is so sick and weak” (Dumase; Site3 FGD 3)

Furthermore, care givers perceived quality of care if they received antibiotics, injectables or other medicines other than the analgesics. Receipt of analgesics alone, whether appropriate for the child’s condition was usually cited as a discouraging factor that affects subsequent utilisation of child healthcare. Assertions like ‘*Getting only Panadol*, *you just feel like you have wasted your time and were better off buying from local shops*’ were common among caregivers in all FGDs.

### Waiting time at a health facility

Waiting time emerged as a prominent theme cited in all FGDs and the majority of KII. Divergent opinions emerged on reasons attributable to long waiting times. Health workers emphasised long queues and a critical shortage of staff to cater for the demand for under-five child and adult health services on a day-to-day basis, as implied by one health worker:

“…the population is growing and so is the demand for health services. Right now our capacities cannot meet the demand. We sometimes keep mothers from early morning up to afternoon, which is a long time, it is a demotivating factor. They do not like to wait for long” (KII 1; Community Health Nurse)

Similarly, health workers indicated that staff shortages were a critical factor contributing to long waiting time and compromised quality of child healthcare:

“Like today I was alone against 200 or more in the under-five Out-Patient Department not mentioning the numbers in the adult one that I also had to attend to. In this way, I cannot give people all the attention they deserve and need” (Vivian; Medical Assistant)

However, caregivers also emphasised late opening of health facilities as contributing to long waiting times, expressing frustration with late opening times of health facilities in the morning and re-opening after lunch break, as observed in an excerpt below:

“They have indicated on their signpost that they will be starting work at 7:30 AM but we wait till 9 AM before they start working. This disappoints people” (Palisha; Site3 FGD 2)

Furthermore, discussions also implied that at certain times of the day, health facilities are seemingly inaccessible. For example caregivers cited that visiting health facilities in the afternoon often attracts the discontent of health workers.

“sometimes a child wakes up well but then gets a sudden fever around noon but when you go to the hospital they say ‘sick people do not come in the afternoon, they come early’. And you do not know what to do so you keep on waiting. They will eventually assist but very late and during which time they don’t undertake proper examination” (Hilda; Site2 FGD 1)

### Distance to health facility

The distance to health facility was cited as an important determinant of care-seeking of under-five health services in all FGDs in Site 3 whereas it emerged in only three of the five other FGDs in sites 1 and 2.

“…there is a long distance for people to get their sick child to the nearest public health facility. Walking is the commonest means to the hospital and if people think of the distance they have to travel, they wait in most cases to the detriment of the ailing child” (Bettie; HSA KII)

Whereas distance to a health facility was viewed of lesser dominance in sites 1 and 2 relative to site 3, in both settings, concerns about travelling to a health facility in the event of a childhood illness at night were more prominent for security reasons.

### Cost of healthcare and household income

Cost of healthcare and inadequacy of household income emerged as two inseparable factors that are barriers to accessing child health services. Due to the predominant use of public health facilities that provide free health services at the point of use, the cost of healthcare did not emerge as an explicit theme suggestive of its deterrent effect to access of under-five child health services. However, in almost all FGDs, participants reported being averse to use of private health facilities due to household financial inadequacy, hence commonly use public health facilities, despite the challenges.

In addition to cost of healthcare, other associated costs such as transport to the facility; and purchasing medicines, as is the case when prescribed medicines are out of stock at the health facility, also emerged. For example some health workers reported experiences where referral is not adhered to as caregivers indicate that they do not have money for transport to enable them travel to the facility where they may have been referred to.

“Sometimes we refer them to central hospital and they say they cannot go because they do not have money for transport” (Emily; Medical Assistant)

### Attitude of health workers

In all FGDs, negative attitudes of health workers were cited as a significant barrier to effective utilisation of under-five child health services. Unfriendly verbal expression, not being adequately attended to when they complain represent some attitudinal challenges that caregivers cited.

“…..you wait for a long time and you see that some health workers are just chatting but when you go to complain they retort that of all the people on that queue, who do you think you are” (Chimwemwe; Site 3 FGD 2).

Nonetheless, some positive attitude from other health workers were cited.

“…it depends with the doctor you find. There are some doctors that treat you well and we are satisfied with the care we receive” (Brenda; Site1 FGD 1)

### Nature of health examination of the sick child

Nature of examination attracted long discussions and emerged as a strong theme in both FGDs and KIIs and appeared to be one of the most important considerations in healthcare utilisation. Caregivers and some health workers reported that the quality or thoroughness of child examination determines their satisfaction with healthcare and this ultimately influences care-seeking decisions in subsequent childhood illness episodes. Caregivers indicated that in many cases, they are dissatisfied with the suboptimal examination usually characterised by: hurried collection of subjective health information of the child from the caregiver; lack of physical examination or laboratory tests and lack of or inadequate explanation pertaining to findings on the child’s illness and associated treatment.

“.....the doctor writes as you are explaining, but before you finish, he has finished writing and is giving you the health passport with a prescription. I think it’s just rude and inappropriate. As we walk back home we share experiences and we realise we have just wasted time and we should just have bought drugs from local shops” (Siphiwe; Site3 FGD 1)

In some cases, while acknowledging the suboptimal examination as a source of dissatisfaction, few caregivers noted that the extreme workload on health workers might contribute to this phenomenon, as one participant remarked:

“…sometimes there are large numbers of patients. There are few free hospitals so due to monetary problems we all rush to these free government hospitals. For instance may be you have 500 patients against one doctor, they get tired and we should understand them. In this way some get appropriate care while others do not” (Martina; Site3 FGD2)

## Discussion

This study aimed at exploring care-seeking practices and barriers to accessing under-five child health services in urban slums in Malawi. The findings indicate multiple dynamics of care-seeking for under-five child health services in urban slums. Care is sought but not always in a timely manner.

Knowledge of home management of common childhood illnesses is generally good albeit further education is warranted on some areas especially those related to irrational use of medicines for diarrhoea, combination of medicines and dosing for antipyretics, and use of medicines left over from a previous episode.

Findings revealed that there are both supply and demand side factors that deter access to under-five child health services among caregivers in urban slums. It is noteworthy that supply side factors were predominantly discussed in relation to the public health system. This is unsurprising as the public health system is the most utilised by the study population.

The cumulative challenge of health system factors in deterring access to under-five child health services has been cited in our study and many studies as a consequence of weak health systems in Malawi and other sub-Sahara Africa countries. In particular these findings are consistent with those of a systematic review of qualitative evidence which, in aggregate terms, postulated that caregivers perceive provision of child health services in public health facilities as insufficient and ineffective; its staff mostly rude and uncaring [29]

Lack of medicines and supplies represented one of the most recurrent factors cited as a barrier of access to under-five child health services. Indeed, various reports have indicated erratic availability of essential medicines and supplies over the years in the Malawi health system [30, 31]. For example, a 60 % stock out of antibiotics for community case management of childhood illnesses was reported [32]. Besides inadequate health financing, various aspects reflecting weak logistics management and information systems have been posited to contribute to inadequacy of essential medicines and supplies at implementation level.

Related to unavailability of medicines and supplies, long waiting time and inappropriate examination emerged as two distinct but related health quality dimensions that are challenges to access of under-five child health services. This renders support to findings from a qualitative study in Zambia that these factors are critical caregiver considerations [33]. Caregivers often cited that health workers do not take time to listen to their explanations about the child’s illness and often take the subjective history hurriedly and sometimes proceed with prescriptions without examining the child or they do so superficially.

Whilst this study did not undertake observation to assess case management of respective children, it appears that health workers did not follow the steps for integrated case management of childhood illnesses as recommended by the Integrated Management of Childhood Illness (IMCI) strategic approach. The widely cited superficial examination or lack of it including hurried gathering of patient history supports this assertion. In a survey conducted in 2009 to assess implementation of health facility based IMCI in Malawi, it was found that performance on many indicators was below target. For instance while IMCI recommends that assessment of the sick child should include checking presence of coughing, diarrhoea and fever, this was undertaken in only 54 % of observed assessments and only 15 % of health workers assessed for presence of child danger signs [34]. The IMCI assessment used overt observations in data collection. The potential for overestimation due to the Hawthorne effect [21] is therefore highly probable. On this basis it is logical to argue that in day-to-day delivery of under-five child health services, quality is further compromised and assertions of caregivers in this qualitative study strongly point to this evidence.

Undertaking a thorough examination of the sick child is often challenged by shortage of health workers who have the pressure to exhaust the long queues. Admittedly, a thorough examination requires more time per child, which in the process entails longer waiting time for other caregivers on the queue. Health workers have provided this challenge as the main reason for suboptimal assessment of the sick child. Nonetheless, it is imperative that management of a sick child is not compromised as it has direct implications on his/her health. Moreover, other studies have demonstrated that a thorough examination is a highly valued dimension of child healthcare quality and caregivers may be willing to wait if this is guaranteed [35].

Negative attitude of health workers in form of verbal expression, represented a theme of recurrence as a barrier. Caregivers lamented that some remarks are dehumanising and hence discourage subsequent use of services. In Malawi, media reports on negative attitudes of some health workers have been reported affirming the existence of the problem on a broader level. Similar findings have been reported elsewhere in sub-Sahara Africa with some authors effectively calling for the need for better engagement between health workers and caregivers [29]. Unmasking specific reasons contributing to such attitudinal challenges is problematic and was beyond the scope of this study. However, other discourse and anecdotal assertions cite the following reasons: psychological pressures of the work environment including too much work with limited resources; lack of interpersonal skills among health workers; weak regulation of professional behaviour; limited supervision, and overall de-motivation by health workers who lack performance incentives. However our findings are in contrast to a study on care-seeking for child health services in Sierra Leone where care givers described nurses as kind hearted and ready to help day and night [36].

Health governance structures at local health facility levels such as hospital ombudsman, complaints and disciplinary committees provide platform for health care users to lodge complaints and health authorities to implement remedial action. However, despite being provided for in policy, these structures are either non-existent or dysfunctional in many facilities. Ensuring functionality of these structures may be the starting point in addressing negative attitudes of some health workers and to improve the health system environment and its responsiveness to clients’ expectations.

Our findings confirm those from previous studies in different contexts that have demonstrated that both direct and indirect costs of healthcare and distance deter access to child health services [33, 37–41]. With particular reference to urban slum residents, the Knowledge Network on Urban Settings (KNUS) posits that cost of healthcare is a more prominent consideration than proximity to health services among the urban poor such as those residing in slums [10].

It is evident that caregivers in urban slums face significant barriers to accessing child health services [15, 42]. Whereas their relatively better off counterparts in urban settings arguably afford to access other child health services such as from private providers, and their rural counterparts have the benefit of other NGO service and a prioritised public health system, urban slum residents are left behind in child health programming. Indeed the urban health programming is very weak probably due to an assumption of the urban advantage.

Essential programmes such as the integrated Community Case Management (iCCM) which entail that community health workers (Health Surveillance Assistants) provide standard treatment for uncomplicated common childhood conditions in the communities are only offered in rural and so called hard-to-reach areas. Considering implementation of these in urban slum settings could potentially improve access to child health services. This would contribute to decongesting urban health centres as less complicated childhood cases would be treated in the community, ultimately reducing workload on health workers and waiting time for those attending at the facility.

The current study’s strength boarders on the rigor involved in data collection and analysis, the choice of an important population group in the context of rapid urbanisation rates and slow economic growth, and the richness in scope of information from policy makers to users. The lack of generalizability for our study findings is a limitation, although one which is common for many qualitative studies. Indeed, qualitative study findings are not thought of as facts that are applicable to the general population, but provide essential descriptions and notions that may be applicable within a specified setting [43]. In the case of our study, the findings can be applied to slum areas in Malawi and similar contexts. Additionally, since the three areas for our study were randomly selected and participant selection used geographically wide areas, would justify application to similar settings beyond the three areas of our study. Moreover some broad under-five child health service delivery areas and barriers to access may provide critical information that may be relevant pointers requiring further investigation to inform general child health programming in developing country contexts.

## Conclusion

In conclusion, this study has highlighted barriers to accessing under-five child health services by urban slum residents in Malawi. Mitigating or removing these barriers is essential promote child health and survival in urban slums. It is therefore important to focus on intensifying health education on importance of timely care-seeking, harmonising effective home management and timely health facility use and strengthening the health system to effectively respond to caregivers’ expectations. This will elicit subsequent demand for under-five child health services when needed, and ultimately contribute to child health and survival.

## Additional files

Additional file 1: FGD guide Healthcare seeking practices. Description: A content guide for conducting Focus Group Discussions for this study. (DOCX 23 kb) Additional file 2: Key Informant Interview guide Healthcare seeking. Description: A content guide for conducting in-depth interviews with key informants for child health policy and child health service provision (serving the urban slum populations for this study). (DOCX 20 kb)

## Abbreviations

CHAM: Christian health association of Malawi
CSDH: Commission on social determinants of health
FGDs: Focus group discussions
KII: Key informant interviews
KNHS: Knowledge network on health systems
KNUS: Knowledge network on urban settings
MDG: Millennium development goals
NHSRC: National health sciences research ethics committee
ORT: Oral rehydration therapy
SDG: Sustainable development goals
