# Supplementary material for: Healthcare seeking practices and barriers to accessing under-five child health services in urban slums in Malawi: a qualitative study
Source: BMC Health Serv Res. 2016 Aug 19;16:410. doi: 10.1186/s12913-016-1678-x (PMC4992285; doi:10.1186/s12913-016-1678-x)
Supplement: Additional file 1: — FGD guide Healthcare seeking practices. Description: A content guide for conducting Focus Group Discussions for this study. (DOCX 23 kb) [file 12913_2016_1678_MOESM1_ESM.docx]

# FOCUS GROUP DISCUSSIONS GUIDE FOR CARE GIVERS ON HEALTH SEEKING BEHAVIOUR, HEALTH SERVICES AND CHILD CARE PRACTICES

**INTRODUCTION AND PARTICIPANT INFORMATION FOR CONSENT**

Hello. My name is ____________ and my colleague is ___________. We appreciate your coming upon invitation. We are conducting a research study as part of doctoral studies. We have chosen three areas in Lilongwe and this area is one of those chosen. The main aim of the research is to find out child health and survival in urban slums. While this study is mainly for academic purposes we will also share what we have learned from community members with policy-makers and encourage them to make appropriate decisions that will help the people of these communities especially pertaining to under-five child health.

We’re interested in learning about what is going on in **[Name of Community]**, especially with regard to the health of under-five children. Many times, people from outside think they know what families are experiencing in this community when they really don’t. To us, you are the real experts, and there’s a lot we can learn from you. So today we would like to ask you a few questions about what people in this community experience and how they handle different situations in pursuing the health of their children.

Your participation in this group discussion is voluntary. You may choose to decline to answer questions that you are not comfortable with. There is no direct benefit for your participation in this study. However as mentioned earlier the findings will be essential to improve child health. We encourage you to participate freely as this is an informal discussion. We wish to assure you that whatever we talk about today is confidential. We intend to record our discussions for purposes of this research. No one outside the research team will have access to this recording. Any of you who wishing to have access to this recording to verify or otherwise is free to do so. Whenever we write a report, we will conceal your identity even when we quote your exact words. If there are any questions you are uncomfortable to answer, kindly let us know

- **Explain the note taking and tape-recording roles**
- **Give a few minutes for answering any questions regarding the interview**
- **Provide ground rules for the discussion**

Finally, we acknowledge how difficult it can be to discuss some of these issues openly. However, your frank responses and discussion will be most helpful to us as we try to really understand these issues. Remember, your answers to our questions will not be considered “right” or “wrong.” They are merely information you will provide based on your experiences, observations, or feelings. Are we all comfortable to proceed? If you agree, we will ask each of you to sign a form giving consent for your participation in this study.

Before we begin, let us introduce ourselves. You could just tell everyone your name and how long you’ve lived in this community.

**CHILD CARE PRACTICES**

1 We are interested to find out the child care practices for children under the age of five

- What are the feeding practices for children in this area from birth to the age of five years? PROBES: - Breast feeding initiation? Colostrum feeding? Exclusive breast feeding? When does complementary feeding start? Frequency of feeding for children? Any traditional concoctions for children?
- What are the child stimulation activities that you perform for children in this community?
- How and where do you dispose of children’s excreta?
- What are this community’s perceptions and practices pertaining to childhood immunizations; including Vitamin A supplementation?
- How do you manage a child with fever at home?
- How do you manage a child with diarrhoea at home?

**CHILDHOOD MORBIDITY**

1. What are the most critical illnesses that children suffer from in this community?

MODERATOR: *List all diseases and problems mentioned*

2. Of the illnesses that you have mentioned, which is the single most important disease or problem that leads to child deaths?

3. Why is this such a big problem in the community?

4. What do you regard as danger signs for child health for which you definitely seek treatment?

**HEALTH SERVICES AND HEALTH SEEKING BEHAVIOR**

1. What do people do when children under 5 years get sick?

- Where do you go to seek health care for children under five years of age?
- How timely do caregivers in this locality respond with care-seeking from a biomedical health provider? Roughly number of days from onset of illness. Discuss factors that determine timely or untimely care-seeking
- How do they undertake home management of common childhood illnesses in this area (*Fever, ARI, diarrhoea*)

2. Can you tell us the major providers of health services in this community? Please indicate any individual, groups, type of facility that you consider as a health service provider entity, whether traditional healer, government or private facility etc?

3. In your opinion, of the ones you have mentioned within your community, who / which facility provides the best care for Under five children? Why?

4. What constitutes good health services for children in this community? Please mention all the attributes you consider to be important for you to classify health services as good

*MODERATOR: Probe until saturation on this subject*

6. If people choose not to take their sick children to a health facility, what do they do instead?

- *Probe on home management of childhood illness, use of traditional healers,*?

7 What problems do you face in accessing and utilizing health services within your area? What are your concerns about under-five child health services in general?

8. We are also interested to know if you have community outreach activities in this community, particularly providing child health services

*MODERATOR: For this question and the next please probe on provision of specific components of Community IMCI as on the reference material given*

9. Are there community health workers that come to work in this community?

- What do they do?
- Where are they based?
- How often do they come here
- Do you pay for their services? How are their services priced?

10. Have there been any programs or initiatives to solve any of the child health related problems in the community?

- What was done?
- Which organization/group did this initiative?
- What do people think about the organization that was involved in this initiative?

11. What should be done to improve the health and survival of U-5 children in this community?

**OTHER SOCIAL ISSUES**

1. We are interested to know who are the key decision makers in households of this community?. Explain particularly with regard to decision making on seeking health services for an U-5 child?
2. In terms of health or other issues, we are interested to know if there are differential treatments between a girl or boy of U-5 years of age? Why?
3. What are the health and social problems that you face in your community especially those that affect under-five children
