# Supplementary material for: Healthcare seeking practices and barriers to accessing under-five child health services in urban slums in Malawi: a qualitative study
Source: BMC Health Serv Res. 2016 Aug 19;16:410. doi: 10.1186/s12913-016-1678-x (PMC4992285; doi:10.1186/s12913-016-1678-x)
Supplement: Additional file 2: — Key Informant Interview guide Healthcare seeking. Description: A content guide for conducting in-depth interviews with key informants for child health policy and child health service provision (serving the urban slum populations for this study). (DOCX 20 kb) [file 12913_2016_1678_MOESM2_ESM.docx]

**INTERVIEW GUIDE FOR KEY INFORMANTS INTERVIEWS IN CHILD HEALTH SERVICE PROVISION AND HEALTH POLICY**

**INTRODUCTION AND INFORMED CONSENT**

*Give the potential participant the Participant Information Leaflet or explain its contents. To sign informed consent form if agree to participate*

**CHILD HEALTH SERVICES IN URBAN SLUMS**

1. How are child health services organized in the urban setting and particularly in the slum communities?
2. One of the major strategies for child health service delivery nationally is the Community Integrated Management of Childhood Illnesses (C-IMCI). I would like to know how this strategy is being delivered within the urban slum communities

- **Probes:** *who are involved; is the community involved in planning, implementation including service delivery. How about issues of coverage of C-IMCI within the urban slum areas? Is the monitoring data disaggregated to capture the slum areas?*
- In particular what specific strategies are in place to promote uptake of child health services for care takers of mothers in urban slum communities? (is education on danger signs for child health part of the intervention package?
- Is the strategy termed integrated Community Case Management (iCCM) implemented within the urban slum areas? What is the scope of implementation?
- Could you explain to me, considering the context of the urban settings, whether you still use Health Surveillance Assistants and/or Community Health Workers in the urban slum settings? If so, how are these cadres operating and how are they being supervised

1. Would you explain to me how the child health and survival structures operate at national, district and community levels?

*Probes: what technical working groups are available? Is the child health and survival working committee operational? How about integration of maternal and child health services? Integration of child health programmes?*

1. In your opinion, do you think there should be special attention in promoting child health and survival among the disadvantaged areas of the urban setting such as the urban slums? Explain?

**Probes**: *issues of social determinants of health such as sanitation, safe water, poverty,*

1. What in particular should be done as an improvement to what is probably already happening or as a new strategy altogether to promote the health and survival of children in urban slums?
2. Other than what you have already mentioned have there been any projects or programs to improve child health services targeting the urban slum communities?

- Which organizations?
- What was the strategy?
- What have been the results

**CHILD HEALTH SERVICE UTILISATION AND DETERMINANTS**

1. What have been your experiences in dealing with urban slum populations in relation to health services in general and child health services in particular?

- *What are common childhood conditions in the urban slums*
- *Child health seeking behaviour, probe on home management, use of traditional healers, any beliefs associated with care-seeking*
- *Child health service utilisation rates*
- *Timeliness in care-seeking in the event of childhood illness (number of days from onset of illness), factors influencing when to seek care from a biomedical health provider*

1. What attributes of a health system do you think are important to care givers of children in slum areas, that influence their decisions to seek care from a health facility?

– **Probes**: *For each health system attribute probe on why it is important, the state of service provision in health facilities serving urban slums, where there are strategies in place to be responsive to provider expectations of a health system*

1. What do you think are barriers to access and utilization of child health services within urban slums? What are your health policy and health service provision concerns, if any pertaining to under-five child health services in urban slums in this area or Malawi?
2. How would you describe the health policy environment for disadvantaged children, such as those residing in urban slums at present?

– **Probes**: *Does the policy environment give any explicit indication to address needs of slum dwellers? Is it likely to?*

**CONCLUSION:**

I appreciate your input into this study. Thank you very much for your willingness to participate. Feel free to ask if you have any questions.

Thank You and Goodbye
